# Eye Movements and Cognitive Functioning in Patients With Schizophrenia Spectrum Disorders: Network Analysis

**DOI:** 10.3389/fpsyt.2021.736228

**Published:** 2021-11-10

**Authors:** Alexander Shmukler, Alexander V. Latanov, Maria Karyakina, Victor N. Anisimov, Marina A. Churikova, Ivan S. Sukhachevsky, Valery A. Spektor

**Affiliations:** ^1^Department of Psychotic Spectrum Disorders, Moscow Research Institute of Psychiatry, The Branch of V. Serbsky National Medical Research Center for Psychiatry and Narcology, Moscow, Russia; ^2^Faculty of Biology, Lomonosov Moscow State University, Moscow, Russia

**Keywords:** eye movements, anti-saccades, neurocognitive deficit, network analysis, schizophrenia spectrum disorders, processing speed

## Abstract

**Background:** Eye movement parameters are often used during cognitive functioning assessments of patients with psychotic spectrum disorders. It is interesting to compare these oculomotor parameters with cognitive functions, as assessed using psychometric cognitive tests. A network analysis is preferable for understanding complex systems; therefore, the aim of this study was to determine the multidimensional relationships that exist between oculomotor reactions and neurocognition in patients with schizophrenia spectrum disorders.

**Materials and Methods:** A total of 134 subjects (93 inpatients with schizophrenia spectrum disorders (ICD-10) and 41 healthy volunteers) participated in this study. Psychiatric symptom severity was assessed using the Positive and Negative Syndrome Scale, the Calgary Depression Scale for Schizophrenia, and the Young Mania Rating Scale. Extrapyramidal symptoms were assessed using the Simpson-Angus Scale, and akathisia was assessed using the Barnes Akathisia Rating Scale. Eye movements were recorded using an eye-tracker SMI RED 500, and cognitive function was assessed using the Brief Assessment of Cognition in Schizophrenia. The statistical analyses were conducted using Minitab 17 Statistical Software, version 17.2.1. Data visualization and additional analyses were performed in the R 4.0.3 environment, using RStudio V 1.3.1093 software.

**Results:** A network model of neurocognitive and oculomotor functions was constructed for the patients. In the full network (which includes all correlations) the median antisaccade latency value is the central element of the oculomotor domain, and the Symbol Coding test, the Digit Sequencing test, and the Verbal Fluency test are central elements in the neurocognitive domain. Additionally, there were connections between other cognitive and oculomotor functions, except for the antisaccade error latency in the oculomotor domain and the Token Motor Task in the neurocognitive domain.

**Conclusion:** Network analysis provides measurable criteria for the assessment of neurophysiological and neurocognitive abnormalities in patients with schizophrenic spectrum disorders and allows to select key targets for their management and cognitive remediation.

## Introduction

Diefendorf and Dodge first reported smooth pursuit eye movement characteristics in patients with *dementia praecox* (1). Since then, there have been numerous studies on the association between eye movements and mental illnesses, including schizophrenia (2–4). The results of these studies suggest that oculomotor characteristics can be considered neurophysiological biomarkers for schizophrenia. Indeed, it has been suggested that disturbed eye movements are “a window into the psychotic mind” (5).

Patients with schizophrenia and schizophrenia spectrum disorders (including schizotype disorder) show decreased smooth-pursuit gain, increased antisaccade error rates and latencies, changes in saccade dynamics, or different fixation patterns when viewing static pictures (2). To study oculomotor disturbances in patients with schizophrenia, a variety of tests and experimental schemes for presenting visual stimuli are used; these tests and schemes reveal the features of the spatio-temporal parameters of the main eye movements (macrosaccades). These saccade parameters are associated with the function of various brain formations involved in a multitude of cognitive processes (6).

A relatively simple test for performing visually guided prosaccades characterizes the processes involved in visual attention (primarily involuntary) (7, 8). The antisaccade test is widely used to assess executive control processes, particularly the inhibition of non-linear reflex movements (7, 9, 10). The test for performing saccades in the Go/NoGo scheme is used to study the working memory and executive control systems. This test is also used to assess attention steadiness and deficits, as well as the ability to suppress irrelevant responses (11, 12). Finally, the memory-guided saccade test is used to evaluate the spatial working memory, as well as the executive control processes that inhibit anticipatory saccades.

Eye movement parameters are often used in cognitive functioning assessments for patients with psychotic spectrum disorders (7, 8, 13). Therefore, it is interesting to compare oculomotor parameters and the cognitive functions that are assessed using psychometric cognitive tests.

It is important to consider that cognitive and oculomotor impairments are transdiagnostic in nature. Because the existing diagnostic categories based on clinical consensus fail to align with the findings emerging from clinical neuroscience and genetics, the new framework of the Research Domain Criteria (RDoC) proposed that both separate diagnoses and the spectrum of psychotic disorders should be considered (14). This was justified by the fact that diagnostic categories based upon presentation signs and symptoms might not reflect the fundamental underlying mechanisms of dysfunction. Schizophrenia spectrum disorders include schizophrenia and schizoaffective disorder, which have significant similarities in cognitive impairments (15). In addition, there is genetic evidence for an association between schizophrenia and schizotypal disorder. Genetic studies have also suggested an association between the severity of schizotypal traits in relatives and schizophrenia symptoms in patients (16, 17). In studies of oculomotor deficits and neurological soft signs, some biological similarity was found between people who scored highly for measures of schizotypy and people with schizophrenia (17). Schizotypal disorder is also included in the category “Schizophrenia, schizotypal and delusional disorders (F20–F29)” (18). All these data suggest that schizotypal disorder should be categorized as a schizophrenia spectrum disorder.

Substantial progress has been made in the research on the general properties of complex systems consisting of many elements. A significant contribution to this research has come from the fields of physics and mathematics. A growing number of medical studies have used “graph theory”, which serves as a mathematical basis for representing various indicators as a network of nodes (or vertices) and the connections between the nodes (edges). The application of network analysis is most suitable for understanding complex systems, which include the interactions between genes, proteins, or disease symptoms, including psychopathological symptoms, and cognitive function (19–22).

The aim of this study was to identify the multidimensional relationships that exist between oculomotor reactions and neurocognition in patients with schizophrenia spectrum disorders. This could help uncover the latent structures of cognitive and oculomotor abnormalities, as well as identify the most central factors. These central factors may indicate the most essential cognitive processes in patients. Identifying such factors could contribute to the development of an individualized approach concerning the choice of treatment targets of the disease. Toward this goal, we applied the network analysis methodology to data collected on eye movement characteristics and cognitive tests from a sample of inpatients with schizophrenia and related disorders.

## Materials and Methods

### Participants

A total of 134 subjects (93 inpatients with schizophrenia spectrum disorders (ICD-10) and 41 healthy volunteers) participated in this study. All the participants gave their written informed consent. The study was approved by the local ethics committee, and it was performed in accordance with the Declaration of Helsinki.

Male and female inpatients (18–60 years, inclusive) eligible for this study included those with schizophrenia, schizoaffective disorder, or schizotypal or delusional disorders, as defined according to the current ICD-10 Classification of Mental and Behavioral Disorders (18). Each diagnosis was confirmed using a Mini-International Neuropsychiatric Interview (M.I.N.I.) (23). Their native language was Russian. The severities of patients' psychiatric symptoms were assessed using the Positive and Negative Syndrome Scale (PANSS) (24). Depression was assessed using the Calgary Depression Scale for Schizophrenia (CDSS) (25), and mania symptoms were assessed using the Young Mania Rating Scale (YMRS) (26). Extrapyramidal symptoms were assessed using the Simpson–Angus Scale (SAS) (27), and akathisia was assessed using the Barnes Akathisia Rating Scale (BARS) (28).

Patients were excluded from the study if they were assessed with more than four points on the P2 (conceptual disorganization), P4 (excitement), P7 (hostility), G10 (disorientation) and G14 (poor impulse control) PANSS items. Patients were also excluded if they had a comorbid dependence on psychoactive substances, a history of traumatic brain injuries with loss of consciousness for at least 10 min, or other organic brain lesions.

The healthy controls had no past or present psychiatric or neurological disorders or a family history of psychiatric disorders, and they were not using psychotropic medications or illicit drugs.

The clinical and demographic characteristics of the participants are presented in Table 1. There were 43 men (46%) in the patient group and 16 men (39%) in the control group. The average ages (±SD) in the patient and control groups were 29.3 ± 8.0 years and 28.6 ± 11.1 years, respectively. Despite there being more men in the patient group, there were no statistically significant differences on this basis. There was also no statistical difference in age between the groups.

**Table 1:** Demographic and disease characteristics of the study participants.

	**Patients**	**Control**
Total	93	41
Male (%)	43 (46%)	16 (39%)
Age (year), mean (SD)	29.3 (8.0)	28.6 (11.1)
Duration of education, years (SD)	13.49 (1.5)	14.27 (1.5)
Schizophrenia, n (%)	40 (43%)	–
Schizotypal disorder, n (%)	16 (17%)	–
Acute and transient psychotic disorders, n (%)	12 (13%)	–
Schizoaffective disorders, n (%)	25 (27%)	–
PANSS scores, mean (SD)	82.2 (13.9)	–
Positive subscale score, mean (SD)	18.3 (3.0)	–
Negative subscale score, mean (SD)	21.7 (3.6)	–
General psychopathology subscale score, mean (SD)	42.3 (6.6)	–
YMRS score, mean (SD)	1.6 (3.8)	–
CDSS score, mean (SD)	4.8 (4.1)	–
SAS score, mean (SD)	0.7 (1.7)	–
BARS score, mean (SD)	0.1 (0.5)	–

Nearly half of the participants (43%) in the patient group had schizophrenia. The mean PANSS score for the patient group was 82.2 ± 13.9, and there were no significant depression or mania symptoms in any of the patients (mean CDSS score −4.8 ± 4.1; mean YMRS score −1.6 ± 3.8). There were also no prominent extrapyramidal symptoms (mean SAS score −0.7 ± 1.7; mean BAS score −0.11 ± 0.5).

### Oculomotor Tests

#### Experimental Setup and Visual Stimuli

For the experiments, the subjects were seated in a sound-attenuated room under photopic adaptation in front of a computer LCD monitor (22”, full HD resolution, with a refresh rate of 60 Hz) that was placed 60 cm away from the subject's head. The head was stabilized using a forehead–chin rest. All visual stimuli were presented against a homogeneous white background. The black cross used as the fixation point (FP) was 0.9° in size and in 99% negative contrast with respect to the background. The green and red circles (0.7° in diameter) used as the cues in the Go/NoGo task were, respectively, in 45% and 78% negative contrast with respect to the background. The same red circle was used as the peripheral stimulus (PS) in the anti-saccade (AS) task.

#### Eye-Movement Recordings

Eye movements were recorded at a sampling rate of 250 Hz using an eye-tracker SMI RED 500 (SensoMotoric Instruments GmbH, Germany) and stored for offline analysis. The SMI BeGaze software package was used to process the video stream of the eye video images and to identify the oculomotor events. A composite algorithm was used to identify saccades and fixations based on an evaluation of eye movement velocities and the dispersion threshold of the oculomotor samples [for detailed description, see (29)]. Before each experimental session, a standard 13-point calibration was conducted several times for each subject and the best case was chosen for the eye movement recording.

#### The Anti-saccade Task

**Figure 1:**
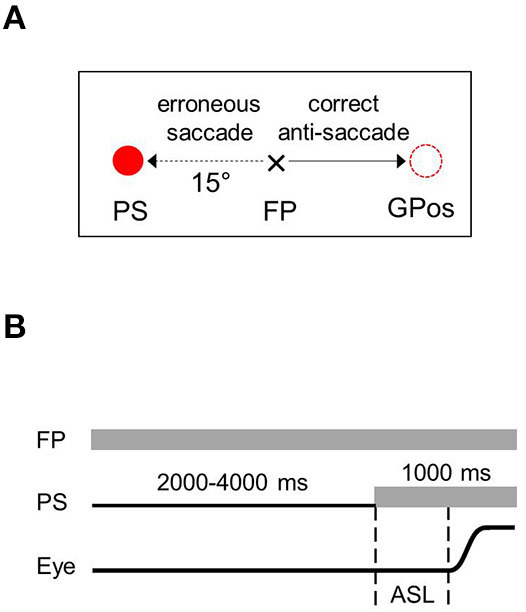
Anti-saccade task. **(A)** Sample trials with the peripheral target occurring to the right of fixation point. **(B)** Time sequences of stimulation and events. FP, fixation point; PS, peripheral stimulus; Eye, schematic eye shift (a saccade); ASL, anti-saccade latency; GPos, gaze position.

In the anti-saccade (AS) task, the subjects were instructed to perform a primary saccade in the direction opposite to a PS to an equidistant position. If the subject performed a primary saccade toward the PS, an erroneous reflexive saccade (anti-saccade error) was counted. Each trial began with the cross (0.9° in size) at the central FP (Figure 1). The subjects were asked to fixate on the FP until the PS appeared after a variable time interval of 2,000–4,000 ms at a peripheral location to the left or right of the FP at an eccentricity of 15°. The subjects were instructed to move their gaze as quickly as possible a distance away from the FP equal to that of the PS but in the direction opposite the PS. Both peripheral locations were used 40 times in a pseudorandom order, which resulted in 80 trials with an intertrial interval of 2000–3000 ms.

#### Go/NoGo Task

**Figure 2:**
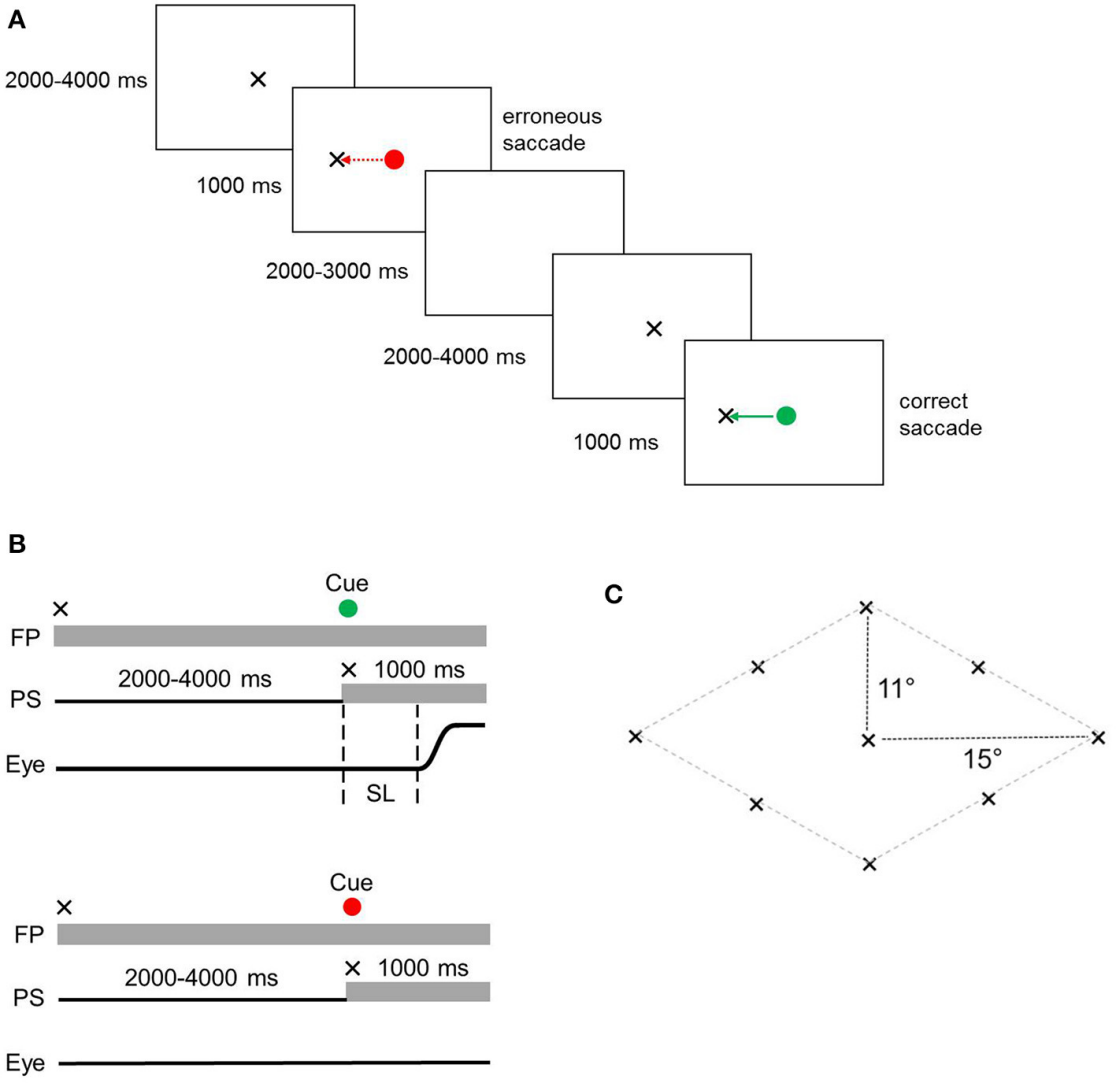
**(A)** Schematic representation of the Go/NoGo task - Sample trials. The NoGo condition - cue is red; Go condition - cue is green. **(B)** Go/NoGo task - Temporal sequences of a representative task trial. Upper – Go condition, lower – NoGo condition. FP, fixation point, PS, peripheral stimulus, Eye, schematic eye shift (a saccade), SL, saccade latency. **(C)** Go/NoGo task - Stimuli locations in the visual field.

In Go/NoGo tests, the subjects were instructed to perform a saccade to the PS as quickly as possible when the cue was green (“Go” condition); conversely, they were required to maintain their gaze on the FP when the cue was red (“NoGo” condition). Each trial began with the cross (0.9° in size) at the central FP (Figures 2A,B). The subjects were instructed to fixate on it during a variable time interval of 2000–4000 ms until the cross jumped to a peripheral location and thereby became the PS. At that point, the cue (green or red circle) replaced the cross in the central location. The subjects were expected to perform a saccade to the PS for the green “Go” condition and to maintain the gaze on the FP for the red “NoGo” condition. If the subjects performed a saccade toward the PS when the signal was red, an erroneous reflexive (non-relevant) saccade was counted. In the reciprocal case, when the subjects maintained their gaze on the FP when the cue was green, an erroneous trial was counted. Each cue color was used 40 times, which resulted in 40 “Go” and “NoGo” trials. The PSs were presented in eight locations in a diamond formation (Figure 2C). Each of the eight locations was used 10 times, which resulted in 80 trials presented in a pseudorandom order with an intertrial interval of 2,000–3,000 ms.

### Cognitive Tests

Cognitive functioning was assessed using the Brief Assessment of Cognition in Schizophrenia (BACS) battery of tests (30), which were validated in Russian (31). There are six sub-tests in this battery, and they assess verbal and working memory, motor skills, verbal fluency, processing speed, and executive functions.

In the *verbal memory (VM) sub-test*, a list of fifteen words was read aloud to each subject, and the subject was then instructed to repeat the words. The procedure was repeated for the five times with the same list of words.

*The Digit Sequencing (DS) sub-test* assesses working memory function. In this test, a series of digits were presented to the subjects in a random order, after which they were instructed to repeat the digits in ascending order. The number of digits in the series (beginning with two digits) increased after every fourth series (ending with eight digits).

*The Token Motor Task (TMT)* assesses motor skills. The subjects were instructed to put as many tokens as possible into a container in one minute by simultaneously picking up one token in each hand and dropping them into the container simultaneously.

In the *Verbal Fluency (VF) sub-test*, the subjects were instructed to say as many words that belonged to a specific category as he or she could in one minute. In the first task, the subjects were instructed to name animals; in the following two tasks, they were instructed to say words beginning with the letters “B” and “S”, respectively.

In the *Symbol Coding (SC) sub-test*, which assesses processing speed, the subjects were instructed to write down the digits that corresponded to specific symbols on a special key that they were shown. Each subject had one and a half minutes to perform the task.

*The Tower of London (TL) sub-test* assesses executive functioning. The subjects were shown two pictures of colored balls on stems, and the task was to count (in their minds) how many ball movements were necessary to put the balls in one picture in the same order as those in the other picture.

### Data Analysis

The raw data were analyzed offline using the SMI BeGaze software package. The task performance accuracy and saccade latency (SL) periods were analyzed. The trials with SL periods <80 ms or >900 ms were discarded from the analysis. During the task performance evaluation, the subjects had made some erroneous saccades or missed the correct saccade, contrary to instructions. The task performance accuracy (PA) was set equal to the number of correct responses divided by the total number of trials.

The statistical analyses were performed using Minitab 17 Statistical Software version 17.2.1. Data visualization and additional analyses were performed in the R 4.0.3 environment, using RStudio V 1.3.1093 software. The mean and SD were calculated to evaluate the statistical characteristics of the SL datasets. The differences between two SL means were estimated using Student's t-test, and the differences between the two variances (SD) were estimated using the *F*-test. An analysis of variances was performed using the Kruskal–Wallis *H*-test. The statistical relationship between the performance accuracy of both tasks and the SL medians was evaluated using the Pearson correlation coefficient. To identify influential (unusual, extreme) observations they were examined using special diagnostic measures (leverage values, residuals, Cook's D, and DFITS) that were evaluated using Minitab Statistical Software package.

## Results

### Eye Movements

#### The Anti-saccade Test

Table 2 represents the oculomotor parameters when performing the AS task in patients and healthy controls. The PA of the AS task varied severely for the patients. Conversely, the PA of the AS task varied slightly for the healthy controls, and they showed higher test performance. The ASLs averaged over all patients significantly exceeds those for the healthy controls.

**Table 2:** Oculomotor parameters from anti-saccade task experiments.

**Group**	**Performance accuracy, median (1^**st**^, 3^**id**^ quartile)**	**Anti-saccade latency Mean ± SD, ms, (N)**	**Error anti-saccade latency Mean ± SD, ms, (N)**
Patients	0.738 (0.619, 0.888)	361 ± 139 (5,262)	241 ± 114 (1,232)
Normal	0.963 (0.913, 0.988)	276 ± 87 (3,033)	228 ± 67 (131)

Four patients were excluded from analysis because of leverage and residual observations. A marginally significant effect of the PA on the ASLs was revealed (H = 55.7, df = 42, *p* = 0.077) in patients. The medians of the ASLs of each patient were significantly negatively correlated with individual PAs (R = −0.564, *N* = 88, *p* < 0.0001). These findings argue for a positive relationship exists between the oculomotor executive processes (AS generation) and the capacity of inhibition control mechanisms that suppress the irrelevant responses.

Four control subjects were excluded from analysis because of leverage and residual observations. No significant effect of the PA on the ASLs (H = 14.6, *p* = 0.331) was revealed in control subjects. Also no correlation (R = −0.002, *p* = 0.992) was revealed between the medians of the ASLs of each control subject and their individual PAs. This result could point the high PAs of control subjects in performing the ASs that are determined by the cognitive control processes function stably and thus a high level of visual-motor integration.

Nearly every patient performed irrelevant (erroneous) saccades toward the PS. Actually such saccades are visually guided and are called anti-saccade errors (ASEs). The median of ASEs proportion for the patients was equal to 10.0 percent with quartile range from 5.0 to 26.3 percent (1^st^ and 3^id^, respectively). The difference between ASLs and ASELs means was highly significant (*p* < 10^−6^), and the variances were highly significant different (*p* < 10^−6^).

Conversely, the healthy subjects exceedingly rarely performed erroneous (reflexive) saccades toward PS. The median of ASEs proportion was equal to 3.8 percent with quartile range from 1.3 to 7.5 percent (1^st^ and 3^id^, respectively). The difference between ASLs and ASELs means was highly significant (*p* < 10^−6^), and the variances were highly significant different (*p* < 0.0001). The PA did not exhibit a significant effect on the ASELs in patients (H = 38.2, *p* = 0.553). Additionally, the medians of the ASELs for each patient did not correlate significantly with the PA (R = −0.148, *p* = 0.178). This result shows no relationship between the reflexive saccades generating and the inhibition of irrelevant reactions.

The medians of the ASELs for the control subjects (six of the subjects did not make any ASEs) significantly and positively correlated with the PA (R = 0.369, *p* = 0.029). This suggests a negative relationship between the weakening of the visual-motor integration, as reflected by ASELs increase, and, presumably, the conflict between the processes of irrelevant reactions inhibition and reflexive oculomotor actions.

#### Go/NoGo Task

Table 3 represents the oculomotor parameters when performing the Go/NoGo task in patients and healthy controls. The PA of the “Go” condition task, estimated as the proportion of the relevant trials (saccade performing when a “Go” signal appeared), varied severely for the patients. Conversely, the PA of the Go condition task varied slightly for the healthy controls, and they showed higher test performance.

**Table 3:** Oculomotor parameters from Go/NoGo task experiments.

	**Go subtask**		**NoGo subtask**	
**Group**	**Performance accuracy, median (1^**st**^, 3^**id**^ quartile)**	**Saccade latency mean ± SD, ms, (N)**	**Performance accuracy, median (1^**st**^, 3^**id**^ quartile)**	**Error saccade latency mean ± SD, ms, (N)**
Patients	0.900 (0.800, 0.975)	409 ± 156 (3,241)	0.600 (0.475, 0.713)	355 ± 154 (1,529)
Normal	1.000 (0.975, 1.000)	379 ± 136 (1,446)	0.800 (0.725, 0.850)	297 ± 81 (335)

In patients a marginally significant effect of the PA for the Go trial on the relevant SLs was revealed (H = 29.9, *p* = 0.054). Moreover the medians of the relevant SLs for each patient were significantly inversely correlated with the PA (R = −0.270, *p* = 0.009). This suggests a slight relationship between the visual-motor integration processes (voluntary saccade generating) and the executive control, with involvement from the working memory.

Four control subjects were excluded from analysis because of leverage and residual observations. A marginally significant effect of the PA for the Go trial on the relevant SLs was revealed (H = 7, 98, *p* = 0,092). Moreover the medians of the relevant SLs for each control subject were significantly inversely correlated with the PA (R = −0.433, *p* = 0.008). This suggests a reasonable positive relationship between visual-motor integration (voluntary saccade generating) and executive control, with involvement from the working memory.

The PA of the NoGo condition task, estimated as the proportion of relevant trials (not performing of a saccade when a “NoGo” signal appeared), also varied severely for the patients. The difference between the relevant SLs (Go condition) and error SLs (NoGo condition) was highly significant (*p* < 10^−6^), although the variances did not statistically differ (*p* = 0.428]).

No significant effect of the PA for the NoGo trial on the error SLs was revealed (H = 30.9, *p* = 0.277). Additionally, the medians of the error SLs for each patient were not correlated with the PA for the NoGo trial (*R* = 0.047, *p* = 0.653). This shows an absence of any relationship between the processes involved in the reflexive saccades generating and the cognitive (executive) control processes with involvement from the working memory.

In control subjects the difference between the relevant SLs and error SLs was highly significant (*p* < 10^−6^), and the variances differed highly significantly (*p* < 10^−6^]). The medians of the error SLs were marginally positively correlated with the PA of the NoGo trials (R = 0.278, *p* = 0.096), indicating the absence of a strong relationship between the processes involved in the performing of visually guided saccades and the cognitive (executive) control.

The PAs of the Go and NoGo trials for the patients correlated slightly, but significantly (R = 0.237, *p* = 0.022). This suggests a slight relationship between the processes involved in each subtest, i.e., there is a common mechanism of executive control that regulates both the performing a relevant saccade (“Go” condition) and the inhibition of irrelevant movements (“NoGo” condition).

Conversely, in control subjects the PAs of the Go and NoGo trials did not correlate (R = 0.110, *p* = 0.515). This shows no relationship between the processes that control the different effects when performing two opposing tasks (executive control and suppression of an irrelevant saccade).

In patients the SLs of the relevant (Go trials) and error (NoGo trials) saccades were moderately correlated with each other (R = 0.654, *p* < 0.0001). In control subjects the SLs of the relevant (Go trials) and error (NoGo trials) saccades were moderately correlated with each other (R = 0.538, *p* < 0.001). These results together suggest a relationship between the visual-motor integration processes involved in each subtest, i.e., a common mechanism that controls the saccades generating.

### Cognitive Tests

The patients performed significantly worse on all the cognitive tests compared to the control group (Table 4). The mean score for the VM test was 43.5 ± 11.2 words. The distribution was nearly symmetrical, with a small deviation toward high values, an asymmetry coefficient of −0.27, and a kurtosis of −0.30. The DS mean score was 18.9 ± 3.8. The test distribution was symmetrical, with a deviation toward high values, an asymmetry coefficient of −0.07, and a kurtosis of −0.58. The TMT average score was 63.7 ± 14.4. The distribution of the TMT was characterized by an asymmetry coefficient of −0.10 and a kurtosis of −0.48. The mean VF test score was 51.4 ± 13.4. The distribution of the VF scores was characterized by an asymmetry coefficient of 0.01 and a kurtosis of −0.15. The SC average score was 49.1 ± 13.5. The distribution was asymmetrical, with a bias toward high values, an asymmetry coefficient of −0.60, and a kurtosis of 1.18. The TL mean score was 16.9 ± 3.8. The distribution was asymmetrical, with a deviation toward high values, an asymmetry coefficient of −1.54, and a kurtosis of 2.94.

**Table 4:** Cognitive tests results in patients and healthy control subjects.

**Cognitive tests**	**Patients**	**Healthy controls**
Verbal memory	43.5 ± 11.2^***^	51.1 ± 6.9
Digit sequencing	18.9 ± 3.8^***^	22.4 ± 2.8
Token motor task	63.7 ± 14.4^***^	75.3 ± 12.0
Verbal fluency	51.4 ± 13.4^**^	60.2 ± 12.6
Symbol coding	49.1 ± 13.5^***^	64.3 ± 9.5
Tower of london	16.9 ± 3.8^***^	19.6 ± 2.4

* *- differences are significant at p < 0.05*;

** *- differences are significant at p < 0.01*;

*** *- differences are significant at p < 0.001*.

### The Network Model

To construct a network, the paired correlations between the different functions in the patients were considered. All the cognitive tests were positively correlated with each other (Table 5).

**Table 5:** Cognitive tests correlations.

**Tests**	**DS**	**TMT**	**VF**	**SC**	**TL**
VM	0.54^***^	0.29^**^	0.42^***^	0.49^***^	0.27^*^
DS		0.39^***^	0.53^***^	0.63^***^	0.41^***^
TMT			0.43^***^	0.53^***^	0.25^*^
VF				0.56^***^	0.50^***^
SC					0.43^***^

* *p < 0.05*;

** *p < 0.01*;

*** *p < 0.001*.

The oculomotor parameters also showed positive correlations, although not all of them were interconnected (Table 6). The ASELs did not correlate with the ASEs or the number of errors in the GnG task (GnGEs); additionally, the GnGLs did not correlate with the GnGEs.

**Table 6:** Oculomotor parameters correlations.

**Parameters**	**ASELs**	**ASEs**	**GnGLs**	**GnG ELs**	**GnGEs**
ASLs	0.31^**^	0.53^***^	0.63^***^	0.51^***^	0.29^**^
ASELs		0.02	0.30^**^	0.33^**^	−0.05
ASEs			0.44^***^	0.33^**^	0.41^***^
GnGLs				0.71^***^	0.07
GnGELs					0.08

* *p < 0.05*;

** *p < 0.01*;

*** *p < 0.001*.

There were negative correlations between the cognitive and oculomotor parameters (Table 7). Not all the parameters showed significant relationships, however. The ASELs did not correlate significantly with the cognitive test scores. Additionally, the TMT did not correlate significantly with the oculomotor parameters, the VM correlated only with the GnGEs, and the TL only correlated with the ASLs. Conversely, the SC correlated significantly with all the oculomotor test results and the DS correlated significantly with four of the six oculomotor parameters.

**Table 7:** Correlation of cognitive and oculomotor parameters.

	**VM**	**DS**	**TMT**	**VF**	**SC**	**TL**
ASLs	−0.17	−0.27^**^	−0.03	−0.21^*^	−0.46^***^	−0.21^*^
ASELs	−0.14	−0.07	0.08	−0.02	−0.08	0.01
ASEs	−0.09	−0.41^***^	−0.04	−0.20^∧^	−0.35^***^	−0.06
GnGLs	−0.09	−0.24^*^	−0.14	−0.23^*^	−0.44^***^	−0.07
GnGELs	−0.06	−0.16	−0.05	−0.24^*^	−0.38^***^	−0.08
GnGEs	−0.24^*^	−0.24^*^	−0.03	−0.02	−0.24^*^	−0.12

∧ *p < 0.1*;

* *p < 0.05*;

** *p < 0.01*;

*** *p < 0.001*.

**Figure 3:**
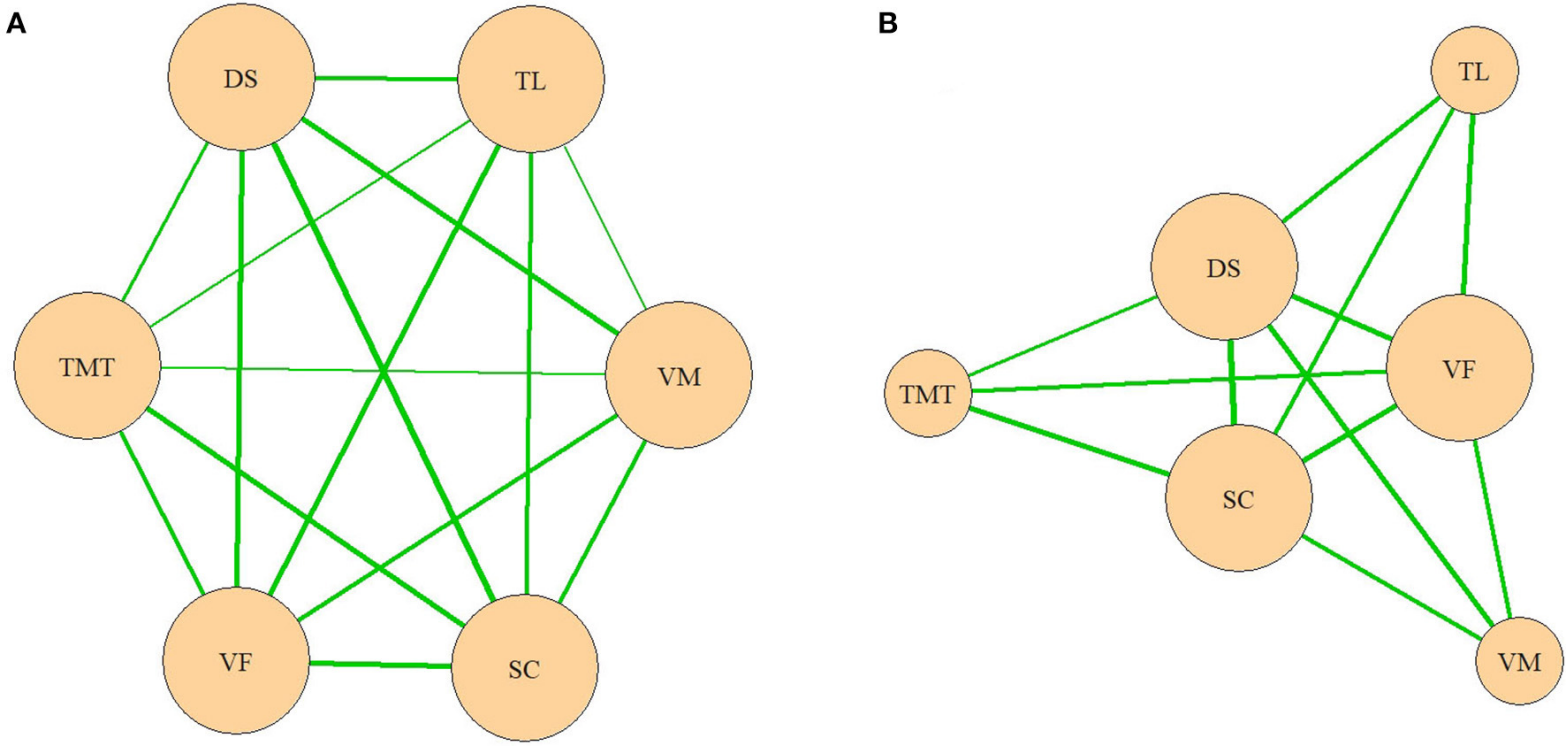
**(A,B)** Network model of neurocognitive functions. VM, test for verbal memory; DS, Digit Sequencing test, which evaluates working memory; TMT, Token Motor Task; VF, Verbal fluency test; SC, Symbol Coding test, which evaluates attention and processing speed; TL, test “Tower of London”, which evaluates executive functions.

Based on these results, a network model of neurocognitive and oculomotor functions can be constructed for the patients. Considering the full neurocognitive network (Figure 3A), all the cognitive functions are almost equally correlated with each other, and the network forms a nearly regular hexagon. However, considering only the correlations >0.4, the working memory (measured by the DS), VF, and processing speed (measured by the SC) have more connections than any of the other parameters (Figure 3B). These three parameters are “central” and have connections to all the other neurocognitive functions, while the results of the VM, TMT, and TL tests connected only to these central functions, but not to each other.

**Figure 4:**
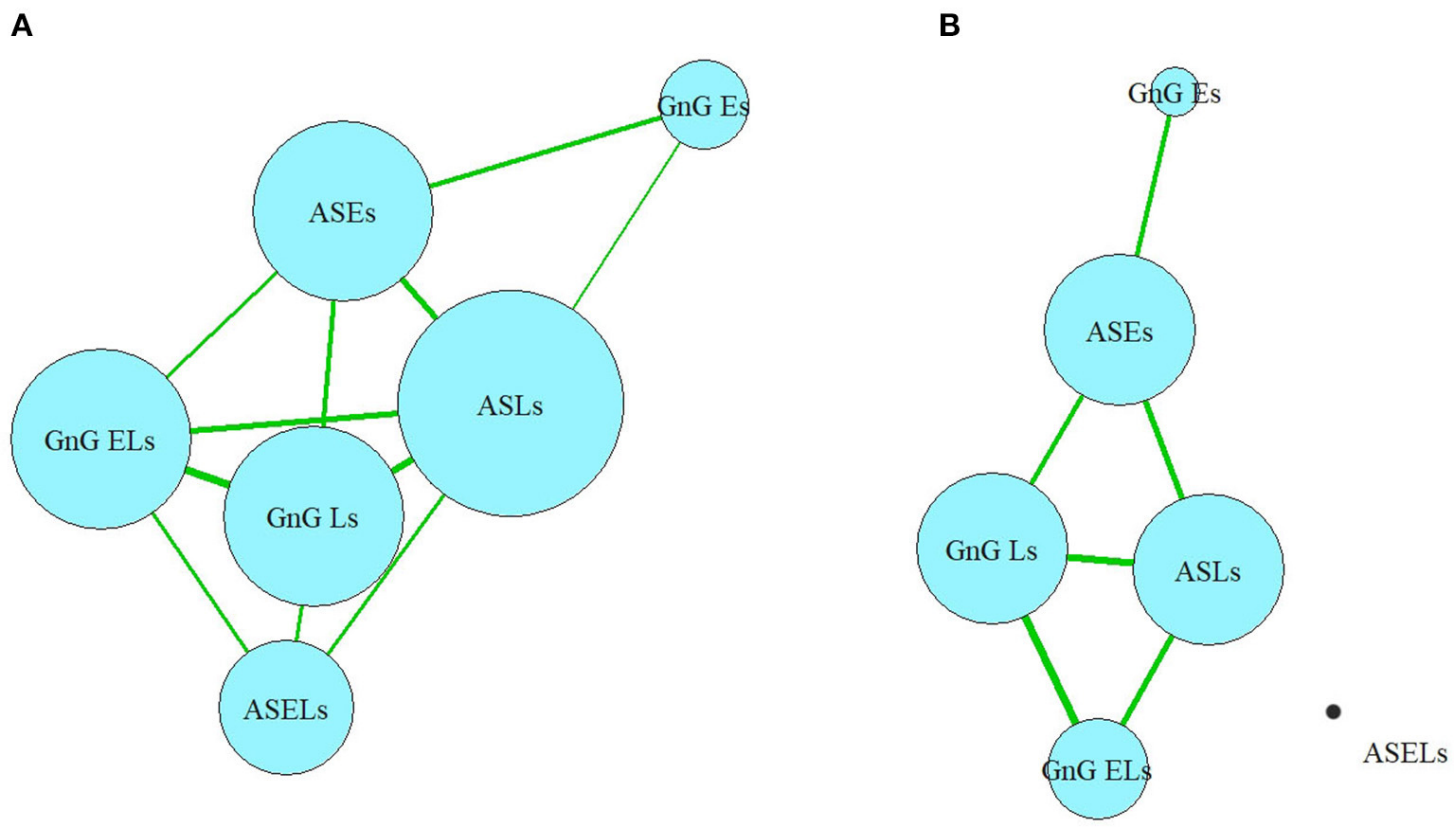
**(A,B)** Network model of oculomotor parameters. ASLs, anti-saccade latencies; ASELs, anti-saccade error latencies; ASEs, anti-saccade errors; GnGLs, latencies in Go/NoGo test; GnGELs, error latencies in Go/NoGo test; GnGEs, errors in Go/NoGo test.

In the oculomotor network, the ASL occupied the central position, with connections to all the other oculomotor functions (Figure 4A). However, when only the correlations >0.4 were considered, it lost its single central position (Figure 4B). In a later network, the ASL, GnG L, and ASE had equal numbers of connections.

**Figure 5:**
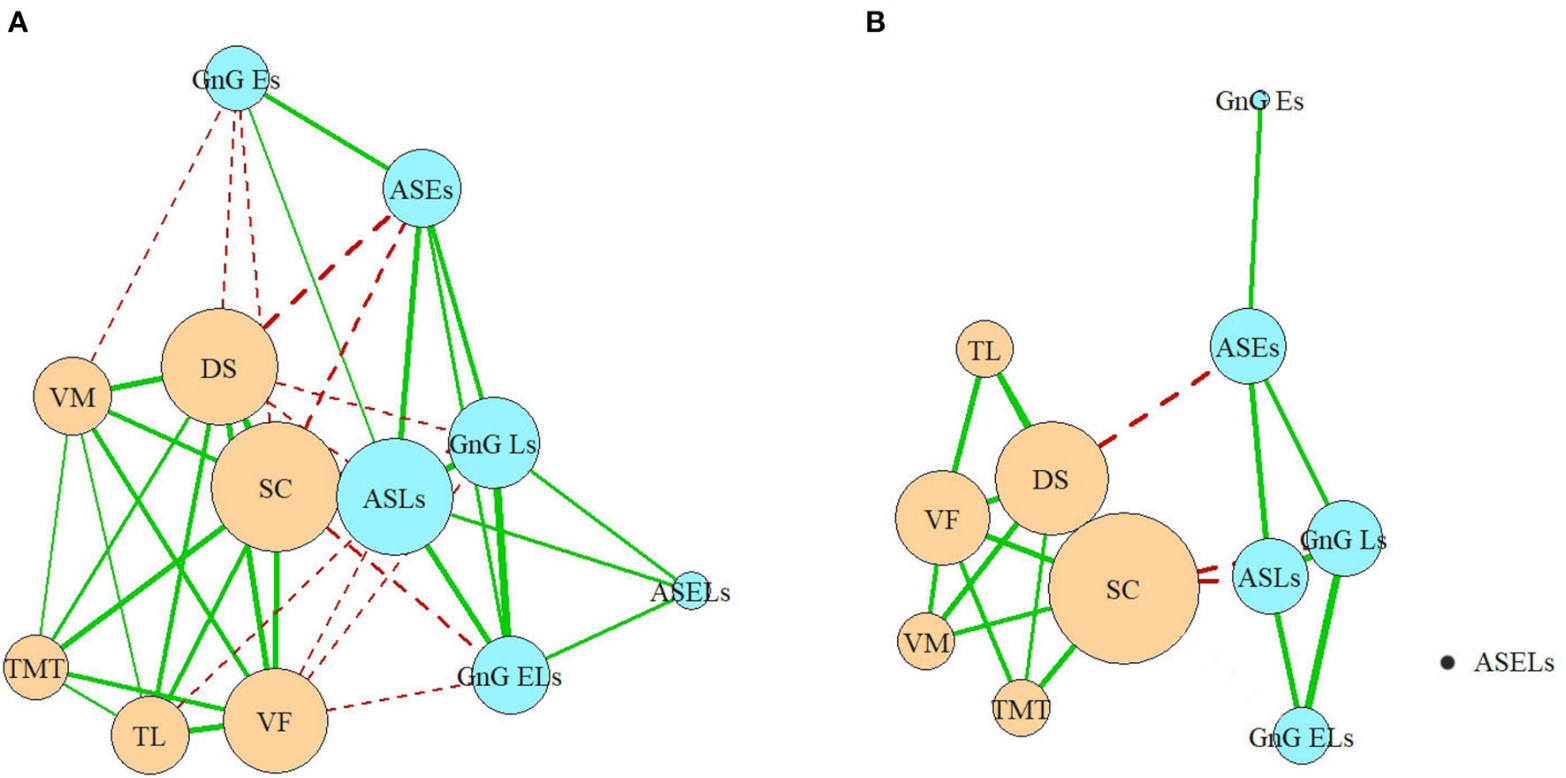
**(A,B)** Full network model. VM, test for verbal memory; DS, Digit Sequencing test, which evaluates working memory; TMT, Token Motor Task; VF, Verbal fluency test; SC, Symbol Coding test, which evaluates attention and processing speed; TL, test “Tower of London”, which evaluates executive functions; ASLs, anti-saccade latencies; ASELs, anti-saccade error latencies; ASEs, anti-saccade errors; GnGLs, latencies in Go/NoGo test; GnGELs, error latencies in Go/NoGo test; GnGEs, errors in Go/NoGo test.

In the full network, which includes all the correlations (Figure 5A), nodes belonging to each domain (cognitive or oculomotor) were generally strongly associated with each other and well separated from the other domain. The ASLs had the most connections of all the oculomotor parameters to the neurocognitive domain, which made it both the central element of the oculomotor network and the central oculomotor element of the full network. In the neurocognitive domain, SC was strongly connected to all the other cognitive nodes and most oculomotor nodes except the ASELs; DS had one less connection (with all oculomotor parameters except ASELs and GnGELs), following by VF (which was connected with ASLs, GnGLs and GnGELs). Negative associations indicated inverted relationships between the domains. That is, the worse the patients' results were in the cognitive tests, the more latencies they showed in the oculomotor tests; the most pronounced connection was with the AS test. However, not all elements of the domains were connected to other domains. For example, ASELs in the oculomotor domain had no connections to the cognitive domain. In the cognitive domain, the TMT had no links to the oculomotor parameters.

Considering only the correlations >0.4, the domains remained relatively separate, and their structures remained relatively preserved. Three of the six oculomotor parameters (ASLs, GnGLs, and ASEs) had one connection each with neurocognitive nodes (Figure 5B). However, the cognitive parameter SC, which measured processing speed, occupied the central position in the neurocognitive domain and became the most central element of the full network. The only other neurocognitive function that had a strong connection with the oculomotor domain was the working memory (measured by the DS), which was connected to the ASEs.

## Discussion

To the best of our knowledge, this is the first report on a network analysis of eye movement characteristics and neurocognitive deficits in patients with schizophrenia spectrum disorders. Liang et al. (21) constructed neurocognitive graphs for first episode schizophrenia patients and major depressive disorder patients and suggested that neurocognitive graphs based on cognitive features are promising for discriminating between patients with schizophrenia or depression and healthy individuals. In the Italian Network for Research on Psychoses study, neurocognition was one of the central elements of a network with links to positive and negative symptoms, depression, extrapyramidal symptoms, and social cognition (32).

In the current study, each neurocognitive parameter has connections to all the other neurocognitive parameters. In the oculomotor domain, the ASLs are identified as the central element, with a greater number of connections to the other parameters in its group. Conversely, numerous parameters (specifically, the ASELs and GnGEs) were determined to be “dead-ends”, having only a single connection or multiple very weak connections. Some of the “network” elements show “circular connections” (VF, DS, and SC for the neurocognitive domain and ASLs, ASEs, GnGLs for the oculomotor domain).

The SC and (to a somewhat lesser extent) DS are central elements in the whole network: the SC connects to all the neurocognitive parameters and five of the six oculomotor parameters (excluding ASELs); the DS connects to all the neurocognitive parameters and four of the oculomotor parameters (excluding the ASELs and GnG ELs). The correlations between the SC and nearly all the oculomotor parameters are more powerful than the correlations between the DS and those same parameters.

Based on our results, we theorize that one of the central mechanisms of functional brain abnormalities in patients with schizophrenia spectrum disorders is the processing speed maladjustment. This hypothesis is consistent with the view that processing speed deficits are one of the key cognitive problems associated with schizophrenia (33–37). Distinct characteristics of schizophrenia appear to predict processing speed subcomponents (36).

Some authors have suggested that processing speed (but not working memory, verbal learning, or executive functioning) is mediated by decreased white matter integrity, which is widespread in schizophrenia (38). Nigg et al. (39) proposed that processing speed has underutilized potential as an endophenotype for psychopathology liability.

A limitation of our study is that the participants had a spectrum of diagnoses (schizophrenia, schizoaffective disorder, schizotypal disorder, and acute and transient psychotic disorders). Presumably patients with different diagnoses may exhibit varying relationships between the studied parameters. A further limitation is the narrow set of investigated features. In the future, it will be necessary to expand the number of studied indicators. It would also be useful to increase the number of neurocognitive tests and add social cognition indicators to the analysis.

In conclusion, our network analysis results provide measurable criteria for the assessment of abnormalities in the neurophysiological and neurocognitive mechanisms in patients with schizophrenic spectrum disorders and allow for the selection of key targets for their management and cognitive remediation. To confirm our conclusions, further research is needed with an increased sample size and added parameters.

## Data Availability Statement

The raw data supporting the conclusions of this article will be made available by the authors, without undue reservation.

## Ethics Statement

The studies involving human participants were reviewed and approved by Local Ethics Committee of V. Serbsky National Medical Research Center of Psychiatry and Narcology. The patients/participants provided their written informed consent to participate in this study.

## Author Contributions

AS contributed to the study design, analysis of the results obtained, and drafting of the article. AL contributed to the study design, conducted the statistical analyses, and took part in drafting of the article. MK conducted the neurocognitive assessment, the statistical and network analyses, and took part in drafting of the article. VA, MC, IS, and VS contributed to the providing of the oculomotor tests. All authors contributed to the article and approved the submitted version.

## Funding

This publication was supported by Future comes today Charity Fund.

## Conflict of Interest

The authors declare that the research was conducted in the absence of any commercial or financial relationships that could be construed as a potential conflict of interest.

## Publisher's Note

All claims expressed in this article are solely those of the authors and do not necessarily represent those of their affiliated organizations, or those of the publisher, the editors and the reviewers. Any product that may be evaluated in this article, or claim that may be made by its manufacturer, is not guaranteed or endorsed by the publisher.
